# Molecular mechanisms of *Tetranychus urticae* chemical adaptation in hop fields

**DOI:** 10.1038/srep17090

**Published:** 2015-12-01

**Authors:** Tara G. Piraneo, Jon Bull, Mariany A. Morales, Laura C. Lavine, Douglas B. Walsh, Fang Zhu

**Affiliations:** 1 Irrigated Agriculture Research and Extension Center, Washington State University, Prosser, WA 99350, USA; 2 Department of Entomology, Washington State University, Pullman, WA 99164, USA

## Abstract

The two-spotted spider mite, *Tetranychus urticae* Koch is a major pest that feeds on >1,100 plant species. Many perennial crops including hop (*Humulus lupulus*) are routinely plagued by *T. urticae* infestations. Hop is a specialty crop in Pacific Northwest states, where 99% of all U.S. hops are produced. To suppress *T. urticae*, growers often apply various acaricides. Unfortunately *T. urticae* has been documented to quickly develop resistance to these acaricides which directly cause control failures. Here, we investigated resistance ratios and distribution of multiple resistance-associated mutations in field collected *T. urticae* samples compared with a susceptible population. Our research revealed that a mutation in the cytochrome b gene (G126S) in 35% tested *T. urticae* populations and a mutation in the voltage-gated sodium channel gene (F1538I) in 66.7% populations may contribute resistance to bifenazate and bifenthrin, respectively. No mutations were detected in Glutamate-gated chloride channel subunits tested, suggesting target site insensitivity may not be important in our hop *T. urticae* resistance to abamectin. However, P450-mediated detoxification was observed and is a putative mechanism for abamectin resistance. Molecular mechanisms of *T. urticae* chemical adaptation in hopyards is imperative new information that will help growers develop effective and sustainable management strategies.

As a flavoring and stability ingredient in beer, hop (*Humulus lupulus*) is an economically important crop in the Pacific Northwest (PNW) of the United States. The U.S. hop industry is concentrated in the three PNW states, Washington, Oregon, and Idaho, which represent over 99% of the nation’s^1^ and 30% of the world’s^2^ hop acreage in 2013. The preliminary production of the U.S. hops crop was valued at $249 million in 2013^1^. Hop is a dioeciously perennial specialty crop that is planted in female monoculture^3^. Hops bloom in the PNW initiated by long days, and un-pollinated flowers develop into cones that ripen between mid-August to mid-September. The commercial products from hops are resin and hop oil extracted from the lupulin gland of the hop cone^4^. In the State of Washington hops are only grown commercially in the Yakima Valley including three distinct growing areas: the Moxee Valley, the Yakama Indian Reservation, and the Lower Yakima Valley. Each of these areas are within a 50-mile radius (80  kilometers) of each other in shrub-steppe habitats characterized by low annual winter precipitation and hot dry summers. Climate, experienced growers, established infrastructure, and modern drip irrigation techniques enhance optimal hop production in the Yakima Valley and make it among the most productive hop growing regions in the world^5^. In 2013, Washington growers produced 79% of the U.S. hops crop^1^.

Integrated pest management strategies have been developed to optimize production of high-quality hops^6^. To date, several plant pathogens and arthropods have been reported as pests of hops in the PNW^6^. Among these, *Tetranychus urticae* is the most serious and prevalent arthropod pest in warmer dry climates^6^,^7^ and efficient control of this pest is a priority for the hop industry. In most situations, hops growers apply various acaricides to suppress *T. urticae* populations. Grower records indicate that up to nine pesticides were used over the course of the hop growing seasons each year in 2012 and 2013^8^. These pesticides include several classes of acaricides with different modes of action. Unfortunately *T. urticae* has been documented to quickly develop tolerance and resistance to these acaricides, which has been linked to control failures^6^. The accelerated development of resistance in *T. urticae* is not only due to the extensive exposure to acaricides, but is also exacerbated by the biology of *T. urticae*, including an extremely short life span with relatively high fecundity, and arrhenotokous reproduction^9^,^10^. As its name implies, *T. urticae* is able to produce webs from silk glands located at each palp^11^. The webbings made by *T. urticae* may work as a shelter to avoid pesticide exposure and protect it from other life-threatening conditions including wind, rain, and natural enemies^12^. Moreover, *T. urticae* undergoes diapause in soil, tree bark, ground cover and dried leaves when decreasing temperature, photoperiod, and decline in the quality of food supply occur due to plant senescence^13^. Diapause facilitates *T. urticae* adaptation to the agroecosystem and offers a refuge to escape pesticide exposure. Indeed, the two-spotted spider mite has been reported to be the world’s most resistant arthropod; this species has been found to be resistant to 94 unique insecticide/acaricide active ingredients in 468 documented cases worldwide^14^,^15^.

The mechanisms of pesticide resistance exhibited by arthropods typically evolve along several trajectories, including behavioral avoidance^16^, decreased cuticular penetration^17^, enhanced sequestration or metabolic detoxification^18^, and target site insensitivity^19^,^20^,^21^. Among these, target site insensitivity to acaricides in *T. urticae* have been investigated extensively^22^,^23^. For example, several mutations in the acetylcholinesterase (*AChE*) gene have been documented in organophosphate resistant *T. urticae* strains from Europe and Korea^24^,^25^. Two mutations were identified in the Glutamate-gated chloride channel (*GluCl*) genes that are correlated with abamectin resistance in *T. urticae* populations^23^,^26^. Studies reported that resistance to bifenazate commonly used for *T. urticae* control was tightly linked to multiple mutations at the Quinol oxidation (Q_o_) site of mitochondrial cytochrome *b* (*cytb*)^27^,^28^. Pyrethroid resistance in *T. urticae* has been associated with several amino acid substitutions in the voltage-gated sodium channel (*VGSC*) gene^29^,^30^. Additionally, recent studies revealed that a mutation on the chitin synthase gene may contribute to resistance to etoxazole^31^, hexythiazox, and clofentezine^32^ in *T. urticae*.

In order to design the most effective and sustainable *T. urticae* management strategy, our long-term goals include revealing the mechanisms underlying the chemical adaptation of *T. urticae* in the field. We initially calculated the baseline concentration response curves of *T. urticae* population susceptible to three acaricides: abamectin, bifenazate, and bifenthrin. We chose these three compounds because they are currently the most commonly used acaricides for *T. urticae* control in hopyards according to the spray records we investigated (Fig. 1). Recently, field control failures with these acaricides have been observed in the Yakima Valley of Washington State^8^. We collected 31 *T. urticae* field populations from hopyards in the Yakima Valley during summer 2013 (Fig. 2) and evaluated the acaricide resistance levels in most of these populations compared with a susceptible strain. We also investigated the distribution pattern of resistance-associated target site mutations in these field collected *T. urticae* populations. Finally, the relative expressions of several detoxification-related P450 genes in field *T. urticae* populations were compared with that of the susceptible population.

## Results

### Baseline toxicity of the lab susceptible population

To establish baseline levels of susceptibility and discriminating concentrations for three acaricides, their toxicity was first evaluated in the susceptible *T. urticae* population. All acaricides tested caused 100% mortality of spider mites at concentrations equivalent to the field rates of 23 mg a.i./L (abamectin), 899 mg a.i./L (bifenazate) and 120 mg a.i./L (bifenthrin). Probit analysis showed that the dose responses of susceptible *T. urticae* to these three acaricides are significantly lower than field rates (Table 1).

### Acaricide resistance levels in field populations

The toxicities of abamectin and bifenazate were assessed for *T. urticae* populations collected from 13 and 12 hopyards, respectively (Tables 2 and 3). In the bioassays with abamectin, the LC_50_s ranged from 1.36 to 26.05 mg a.i./L and the resistant ratios (RRs) compared with the susceptible strain varied from 5.96 to 114.25 (Table 2). Low resistance levels (RR < 10) were observed in 10.5% of the surveyed populations, 10.5% had high resistance (RR > 100), and the majority of the surveyed populations (79%) exhibited moderate resistance (RR = 10–100) to abamectin (Fig. 3A). The RR of the *T. urticae* population in the organic hopyard (Granger 2) compared with the susceptible population was 11.23, which is the 3^rd^ lowest resistance among surveyed populations and the highest level of mortality (100%) at the field rate. Samples collected from the Granger 4 hopyard showed the lowest resistance ratio (RR = 5.96) compared with the susceptible population. There were three 1^st^ year (baby) hopyards (Prosser 3, 4, and 5) surveyed in 2013. The RRs of samples collected from these baby hopyards ranged from 21.80 to 114.25, exhibiting a moderate to high degree of resistance (Table 2). There were multiple collections from certain hopyards (Prosser 2, 3 and 4) during the course of summer 2013. Specifically, six collections were taken from the Prosser 2 hopyard starting from middle of June till just prior to harvest in late August during which abamectin was applied twice^10^. The RR increased 6-fold from the middle of July to mid-August (Table 2). The RRs in samples collected from Prosser 3 and 4 increased 1.7-fold and 2.3-fold in four and five weeks, respectively. The highest resistance level to abamectin was recorded at the Prosser 4 (RR = 114.25) (Table 2).

In the bioassays with bifenazate, the LC_50_s ranged from 3.93 to 78.97 mg a.i./L and the RRs varied from 4.79 to 96.30 (Table 3). Populations exhibiting low resistance levels (RR < 10) accounted for 37.5% of the populations surveyed, and 62.5% of the populations exhibited moderate resistance (RR = 10–100) to bifenazate (Fig. 3B). The lowest RR to bifenazate, 4.79, was recorded from the samples collected from the organic hopyard (Granger 2). The RRs of samples collected from the 1^st^ year hopyards showed low to moderate level of resistance (Table 3). The highest RR to bifenazate was observed in the sample collected from Granger 3 (RR = 96.30) (Table 3). Due to the limited number of collected *T. urticae* individuals in four populations, only the discriminating dose of bifenazate was evaluated (Table 3).

### Evaluation of target site mutations

The occurrence of 16 mutations in four target genes, *GluCl1* and *GluCl3* (target of abamectin; Fig. S1), *cytb* (target of bifenazate; Fig. S2), and *VGSC* (target of bifenthrin; Fig. S3), was examined in *T. urticae* field populations by direct sequencing of PCR products. By visual examination of sequencing chromatographs at the mutation sites, we could identify samples that contained wild-type, resistant, or both alleles. The combination of mutations in field *T. urticae* populations collected from PNW hopyards exhibited a unique pattern (Table 4). Only two mutations, G126S and F1538I, in *cytb* and domain III of *VGSC*, respectively, were identified (Table 4). There were no mutations observed in *GluCl1, GluCl3*, and other region of *cytb* and *VGSC*.

#### No mutations observed in Glutamate-gated chloride channel genes

Inhibitory Glutamate-gated chloride channels (GluCls), members of the cys-loop ligand-gated ion channel (cysLGIC) superfamily, are extrajunctional or postsynaptic receptors found in muscle or neural ganglion of most protostome phyla including Chelicerates such as *T. urticae*^23^,^33^. The genome of *T. urticae* contains six orthologous *GluCl* genes^23^. Previous studies revealed that two mutations in two different GluCl channel subunits, GluCl1 and GluCl3, were related to abamectin resistance in *T. urticae*^23^,^26^. Thus we designed primers to sequence the fragments containing these two mutations (Fig. S1) from susceptible and all hop field populations of *T. urticae*. Surprisingly, there were no mutations identified from the samples tested (Table 4), suggesting target site insensitivity-mediated resistance is not the mechanism leading to the abamectin resistance that we observed in *T. urticae* field populations.

#### Identification of mutations in the cytb gene

Recent studies suggested that bifenazate resistance was closely correlated with mutation(s) in the mitochondrial cytb^27^. A combination of at least two cd1 helix mutations in the Q_o_ pocket (G126S and I136T or G126S and S141F) and one mutation in the ef helix of Q_o_ pocket (P262T) were linked with a high level of bifenazate resistance in *T. urticae*. We sequenced an 828 bp fragment of the *T. urticae cytb* gene, which included the G126, I136, S141, D161 and P262 sites (Fig. S2) that have been demonstrated to confer bifenazate resistance in *T. urticae*^27^. One amino acid substitution, G126S, was detected in *T. urticae* field populations. 35% of field samples field samples contained only the resistant allele, 20% contained both alleles (G/S) and 15% only the susceptible allele (G) (Table 4; Fig. 4A). Since the G126S mutation alone only causes low to moderate bifenazate resistance^27^, this result is consistent with the bifenazate resistance phenotype observed (Table 3).

#### Identification of mutations in the voltage-gated sodium channel gene

The voltage-gated sodium channel (VGSC) is an integral transmembrane protein that is responsible for the rapidly rising phase of action potentials on the neuronal membranes. Due to its essential role in electrical signaling, VGSC is the target of several neurotoxins, including pyrethroids and DDT^34^. Many amino acid substitutions associated with pyrethroid resistance in arthropods are located in transmembrane segments 4–6 of domain II (IIS4-IIS6) including M918 (super *kdr*), L925, T929, L932, V1010, L1014 (*kdr*), and L1024^30^,^34^,^35^,^36^. One mutation within the intracellular inter linker connecting domains II and III (A1215D) and one mutation in domain III (F1538I) were detected in a highly bifenthrin resistant *T. urticae* strain from Greece^29^. Thus we amplified three fragments of the *VGSC* from the domain II, II-III inter linker, and domain III regions (Fig. S3). We identified only one amino acid substitution, F1538I. It was observed in 16 out of 24 field samples tested (66.7%), 12 of which contained both alleles (F/I) and 4 of which were only contained the isoleucine substitution (I) (Table 4; Fig. 4B).

### Cytochrome P450-mediated metabolic detoxification

Besides target site insensitivity, cytochrome P450-mediated detoxification had been shown to be one of the most important mechanisms in acaricide resistance of *T. urticae*^37^,^38^,^39^. The genome of *T. urticae* contains 86 P450 genes. We examined the relative expression of three P450s, *CYP385C4, CYP389A1*, and *CYP392D8*, belonging to the CYP3, CYP4, and CYP2 clans, respectively. We chose these three P450s because they have been shown to exhibit more than two-fold up regulation after switching host plants and their expression patterns have been linked to acaricide resistance in *T. urticae*^9^. The expressions of these three P450s in five field populations from five major locations were compared with their expressions in the susceptible population. As shown in Fig. 5, *CYP385C4* had significantly higher expression in all five field populations. However, this increase in expression was not large (less than two-fold). *CYP389A1* only showed significantly higher expression in the Prosser 2 population. The expression of *CYP392D8* was strikingly higher in all five field populations, exhibiting levels 5 to 40-fold higher than the susceptible strain. It indicates that *CYP392D8* may play an important role in acaricide resistance of *T. urticae* populations in hopyards.

## Discussion

Due to a very short residual effectiveness, abamectin has become the predominant acaricide applied to control *T. urticae* outbreaks in August as the hops near harvest. Annually, approximately 98% of the hop acreage in Washington is treated with abamectin at least once and 80% is treated at least three times. The widespread use of abamectin on hops raises the distinct possibility of control failure as a result of resistance. From sampling the same hopyard over multiple time points in the same season, we found increasing levels of abamectin resistance, suggesting selection pressure from abamectin applications was driving increasing resistance. For instance, multiple collections in the Prosser 2 hopyard showed that the RR to abamectin increased 6-fold from the middle of July to mid-August (Table 2). The highest LC_50_s to abamectin were recorded in the Moxee 1 and Prosser 4 *T. urticae* populations (Table 2). However, Moxee 1 had only two acaricide applications during 2013^8^. The possibility for the reported highest abamectin resistance ratio in Moxee 1 could be the entire application history of abamectin in this field that remains unknown. There may have been high abamectin selective pressure over multiple overwintering populations in this field prior to 2013. The high level of abamectin resistance in the Prosser 4 population was also unexpected because this sample was collected from the 1^st^ year baby hopyard^8^. Prior to planting hops, the crop planted in Prosser 4 was Concord grapes. A recent study reported infestation of *T. urticae* in grape yards and high abamectin resistance in 45% *T. urticae* populations from these grape yards in Brazil^40^. Nevertheless, our previous investigation suggested that *T. urticae* is not a pest of grape yards in Washington State^41^ and thus we do not expect the fields have been extensively sprayed with abamectin. However, Bradenburg and Kennedy^42^ reported that wind dispersal was a key factor causing the infestations of *T. urticae* from corn fields to surrounding crops. Thus, the resistant *T. urticae* populations we detected may have been transported from adjacent crops to the 1^st^ year hopyard through wind dispersal.

GluCls together with gamma-aminobutyric acid (GABA)-gated channels and histamine-gated chloride channels (HisCls) are known targets of the macrocyclic lactones, the avermectins (including abamectin) and ivermectins^26^,^33^,^43^,^44^. The point mutation G323D in *GluCl1* was tightly linked to a moderate abamectin resistance (17.9-fold) in the AbaR strain^26^. Two point mutations, G323D and G326E, in *GluCl1* and *GluCl3*, respectively, were identified in a > 2,000-fold abamectin resistant strain^23^. However, there was no mutation on *GluCl* subunits detected in any hop samples (Table 4), suggesting target site insensitivity is not likely the mechanism involved in resistance to abamectin in *T. urticae* field populations. Our results are comparable with a study by Khajehali *et al.*^45^ which found no *GluCl* mutations in 15 *T. urticae* strains collected from rose greenhouses in the Netherlands, although 10 of those strains displayed abamectin resistance. Many recent studies also suggested that abamectin target site mutations are not especially common in *T. urticae* populations worldwide. For example, the G326E was detected in only seven out of 51 *T. urticae* populations sampled from 27 countries and five continents^46^. The G323D mutation was only found in two Greek samples in the same survey^46^. In another study with 25 Korean *T. urticae* populations, only one field-collected *T. urticae* sample contains G323D mutation^47^.

Previous synergism tests and transcriptomic data indicated that additional mechanisms such as enhanced metabolic detoxification by cytochrome P450s may be implicated in the abamectin resistance phenotype^37^,^48^,^49^. A genome microarray analysis revealed several cytochrome P450 genes were up-regulated in an abamectin resistant strain^49^. Further evidence confirmed the function of one of these P450s, CYP392A16, in metabolizing abamectin^50^. Unfortunately, this study was published after the completion of our study, and we did not have enough sample material remaining to test for expression of this gene. However, of the three P450s we did examine in our study, one Clan 2 P450, *CYP392D8*, showed constitutive over-expression in all five field collected samples compared to the susceptible population, indicating its potential function in abamectin resistance (Fig. 5).

Bifenazate is a hydrazine carbazate acaricide that was discovered in 1990 by Uniroyal Chemical and first registered in the state of Washington in 2002^8^,^51^. Because of the quick knockdown and long residual effects on many economically important phytophagous mite species and low toxicity on predatory mites and beneficial insects, bifenazate is widely used as a selective acaricide to control *T. urticae* in hopyards. Our bioassay data demonstrated that the majority of field *T. urticae* populations (62.5%) in hopyards exhibit moderate levels of resistance to bifenazate (Fig. 3B). Our target site mutation screening revealed that a mutation G126S on *cytb* gene occurs in 35% of *T. urticae* populations (Fig. 4A). It should be noted that G126S (GGA to AGA) is the same mutation as described in previous studies^27^,^28^. G126S is the most common substitution on *cytb* gene of *T. urticae* that was identified in several bifenazate resistant populations^22^,^27^,^28^,^45^,^46^. Previous studies showed that mutations on the G137 site in *Saccharomyces cerevisiae* (equivalent to G126 in *T. urticae*) contributed to respiratory-deficiency through affecting stability of FeS^52^,^53^. However, the G126 mutation alone only confers low to moderate level of resistance to bifenazate^28^. In our results, the G126S mutation was observed in populations of Granger 4, Moxee 1 & 2, and Prosser 1, 4 & 5, which all demonstrated low to moderate level of bifenazate resistance (RR = 8.37–23.02 or mortalities at discriminating dose ranged from 90% to 100%) (Table 3), suggesting the resistance phenotypes of these samples are consistent with their genotypes. Other mutations or mutation combinations on *cytb* gene such as P262T, G126S with I136T/S141F that are responsible for high bifenazate resistance with RR > 2778^27^,^28^ were not detected in any of our samples.

**Figure 1:**
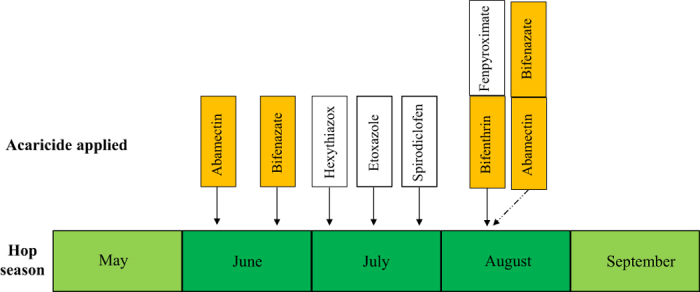
The acaricide spray model at hopyards during hop season in 2013. Several acaricides with different mode of actions were applied to control *T. urticae*. Among them, abamectin, bifenazate, and bifenthrin were commercially important acaricides used in hopyards^10^.

Bifenthrin, a pyrethroid, has been introduced for *T. urticae* control in hopyards since it was registered in 1993^51^. Because of their safety, longevity of residual activity and low cost, pyrethroids are extensively used for pest control, with about a 20% insecticide market share^23^. Unfortunately, ubiquitous resistance to pyrethroids had been broadly reported in various insect populations^19^,^20^,^34^,^36^,^54^. In *T. urticae*, two mutations, F1538I in domain IIIS6 and A1215D within the intracellular inter linker connecting domains II and III were linked with high bifenthrin resistance in a Greek population^29^. The function of the F1538I mutation in pyrethroid resistance has been confirmed^34^,^55^,^56^ while the function of A1215D is still unknown. Another substitution, L1024V in domain IIS6 was reported to play an important role in the fenpropathrin resistance of *T. urticae* from Korea^30^. Pyrethroids are not used very often in the hopyards in PNW since they are linked with subsequent *T. urticae* outbreaks. Therefore we omitted the toxicity evaluation of field collected *T. urticae* samples. However, based on our record of acaricide sprays in hopyards, bifenthrin is still used in August as the hops near harvest^8^ (Fig. 1). Our DNA diagnostic results demonstrated that a mutation in the *VGSC* gene (F1538I) was observed in 66.7% *T. urticae* populations (Fig. 4B). Particularly, F1538I was fixed in four samples collected from Grandview and Prosser 4 & 5, three of which were collected in late August or September (Table 4). Additionally, esterase-mediated metabolic detoxification had also been proposed to confer resistance to pyrethroids in *T. urticae*^57^,^58^,^59^. This result suggests that developing of pyrethroid resistance in hopyards should be of concern.

In summary, *T. urticae* populations in hopyards exhibit a low to moderate level of acaricide resistance. The mechanisms of acaricide resistance in *T. urticae* are likely mediated by a number of different pathways: not only target site insensitivities but also enhanced metabolic detoxification. It is a common phenomenon that multiple genes or mechanisms confer resistance simultaneously to a certain pesticide^18^,^20^,^60^,^61^,^62^,^63^,^64^. Therefore, we plan a genome-wide investigation to identify a more complete set of candidate resistance genes from *T. urticae* populations of hopyards. Our data also suggests that acaricide spray history, neighboring plants, and time of the season are important factors in correctly diagnosing acaricide resistance in *T. urticae.* Developing a baseline effective dose for commonly used acaricides and screening local *T. urticae* populations with resistance-associated molecular markers would be a proactive approach toward *T. urticae* resistance management. Our study reveals a unique phenotypic and genotypic pattern underpinning the chemical adaptation of *T. urticae* in hop fields which will be of assistance in developing diagnostic tools for integrated *T. urticae* management.

## Methods

### Mite samples

The susceptible acaricide naïve *T. urticae* strain (SS) was originally collected from weeds in Montana in 1995 and reared under laboratory conditions without exposure to any pesticides^8^. This population was reared on 2-week-old lima bean plants (*Phaseolus lunatus* L.) at 28 ± 2 °C, 70 ± 5 RH and a photoperiod of 16:8 (L:D) h in an isolated walk-in growth chamber at the Irrigated Agricultural Research and Extension Center (IAREC) in Prosser, WA. Bean plants were grown from seeds (Buckeye Seed Supply, Canton, OH) with medium grade vermiculite (Therm-o-rock West Inc.) soaking in water in the greenhouse. New, healthy, lima bean plants were provided for *T. urticae* and plants were replaced every seven days. To prevent mite migration, the colonies were maintained in 27-L plastic tubs filled with soapy water Huffaker moats^8^.

**Figure 2:**
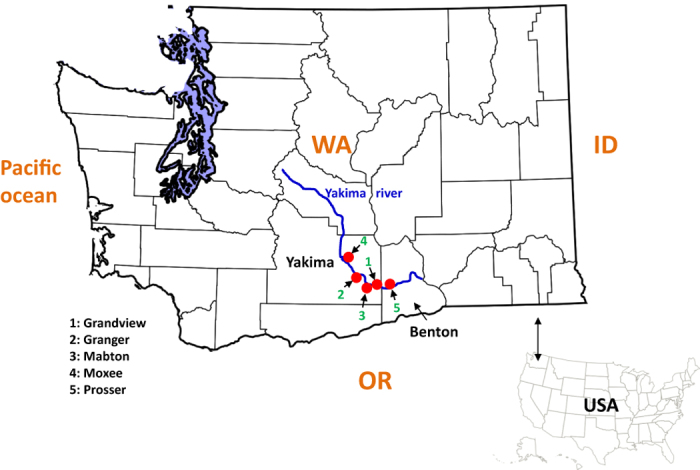
The geographic distribution of 31 *T. urticae* populations collected from 5 major locations. The map of Washington counties was modified from a public domain picture (https://commons.wikimedia.org/wiki/File%3AMap_of_Washington_counties%2C_blank.svg).

**Figure 3:**
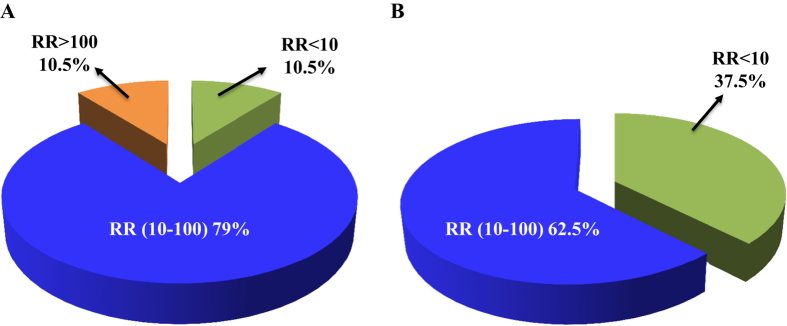
Pie charts illustrating proportions of different levels of Resistant Ratio (RR) for field collected *T. urticae* samples. (**A**) Abamectin resistance; (**B**) Bifenazate resistance. Low level of resistance, RR < 10; moderate level of resistance, RR = 10–100; high level of resistance, RR >100.

**Figure 4:**
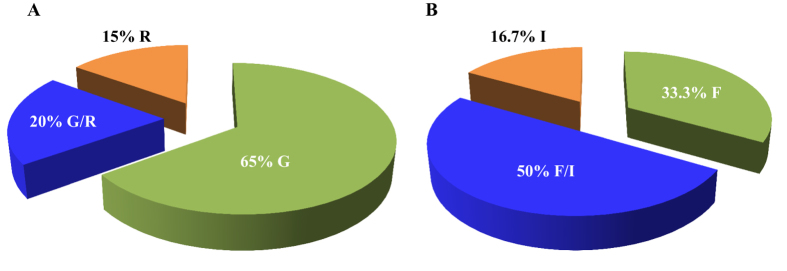
Pie charts showing proportions of resistance associated allele for G126S on *cytb* (A) and F1538I on *VGSC* (B). The colors green, blue, and orange stand for the susceptible allele, double alleles, and resistant allele, respectively.

**Figure 5:**
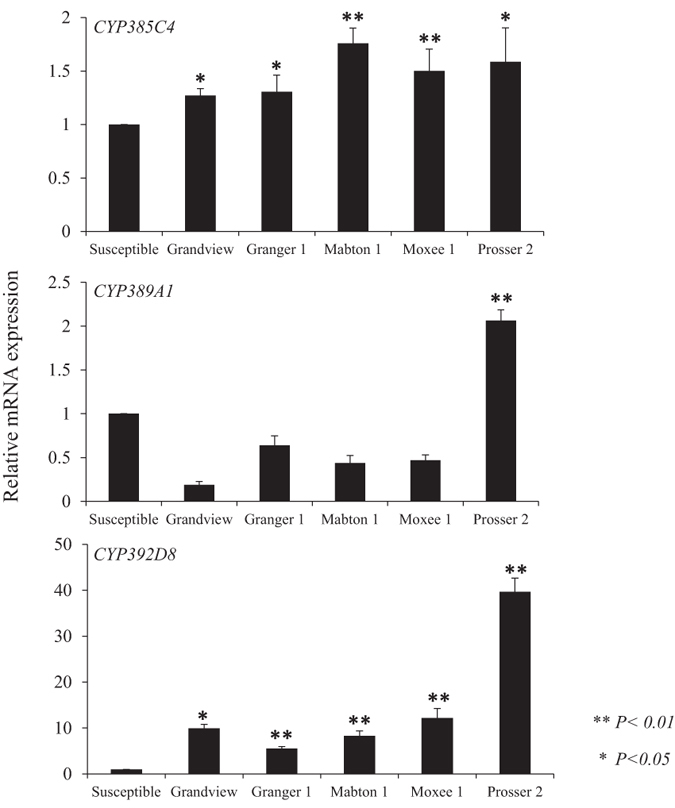
Relative expression of *CYP385C4, CYP389A1* and *CYP392D8* in field *T. urticae* populations compared with that of the susceptible strain. The mRNA levels were quantified by qRT-PCR and normalized with reference genes *Actin* and *RP49*. The data shown are mean + SEM (n = 3). Statistical significance of the gene expression between two samples was calculated using Student’s *t* test (two-tailed distribution). *p-value < 0.05, **p-value < 0.01.

Thirty-one field *T. urticae* populations were collected from commercial hopyards located within the Yakima Valley of Washington State from June to September in 2013. There were five major locations: one sample was collected in Grandview, WA (46°15′13″N 119°54′36″W), eight in Granger, WA (46°20′40″N 120°11′29″W), four in Mabton, WA (46°12′42″N 119°59′47″W), two in Moxee, WA (46°33′23″N 120°23′14″W), and 16 in Prosser, WA (46°12′25″N 119°45′56″W) (Fig. 2; Tables 2, 3, 4, [Table t2], [Table t2], [Table t3], [Table t3], [Table t3], [Table t3], [Table t4]). The samples collected from the same location at different times were treated as different populations. Mite-infested hop leaves were stored in a plastic bag and transported to the lab in a cooling box within a few hours of collection. Spider mites were identified under a dissecting scope according to morphological characteristics^13^. Approximately 50–100 adults were stored in 95% ethanol for genomic DNA extraction. About 300 adult *T. urticae* from each of five major locations listed in Table 4 were also stored in RNAlater® (Sigma-Aldrich, Saint Louis, MO) for RNA extraction. Remaining mites were used for bioassays directly. Because three samples had a low number of mites (Table 4), we reared them on lima bean plants in an isolated walk-in growth chamber for one month to increase population size before sampling them for DNA extraction and bioassays.

### Bioassays and data analysis

Leaf disc bioassays were used to estimate the LC_50_ (lethal concentration required to kill 50% of the individuals in a population) of abamectin and bifenazate for lab susceptible and field spider mite populations. The method followed that of Knight *et al.*^65^. Briefly, ten female adult spider mites were placed on the back of a bean leaf disc (2 cm diameter) with a fine brush. Two leaf discs were arranged on water-saturated cotton (4 cm × 4 cm) in a single petri dish (9 cm diameter, 1.5 cm height; Alkali Scientific, Pompano Beach, FL). The water-saturated cotton was pushed up against the perimeter of the leaf disc to prevent mites from walking off the disc^65^. Two commercially formulated acaricides for leaf disc bioassay are Epi-mek® 0.15 EC (2% a.i. Abamectin, Syngenta Crop Protection) and Acramite® 50WS (50% a.i. Bifenazate, Chemtura Agro Solutions). The recommended field concentrations for these two acaricides are 23 mg a.i./L and 899 mg a.i./L, respectively. The field rate solutions were prepared in the lab using commercial formulated acaricides and distilled water. These solutions were serially diluted in distilled water for 4–7 concentrations ranged from 0.1–67 mg a.i./L and 0.44–889 mg a.i./L for Epi-mek® and Acramite®, respectively.

The sticky tape method was used to estimate the LC_50_ to bifenthrin for the lab susceptible strain because pyrethroids are shown to have repellent effects on mites^66^. In this method, ten female adult spider mites were placed dorsal side down on a strip of double-sided sticky Scotch® tape (3cm × 1.2 cm) stuck on a glass slide (7.5 cm × 2.5 cm). The commercially formulated bifenthrin was Bifenture® EC, a pyrethroid provided by United Phosphorus (25.1% a.i. Bifenthrin). These bifenthrin solutions were serially diluted in distilled water for 4–7 concentrations ranged from 6–120 mg a.i./L.

Leaf discs or glass slides were treated topically with 2 ml of acaricide solutions with a Potter spray tower (Burkard Manufacturing, Richmansworth, Herts, UK)^67^. The tower was calibrated to deliver 1.1 kg/cm^2^ which allowed 2.0 ± 0.1 mg/cm^2^ spray fluid. Each bioassay consisted of 4–7 acaricide concentrations with 4–6 replicates for each concentration. The spider mites exposed to distilled water in the Potter spray tower were used as the non-treated control. The treated leaf discs or glass slides were maintained at 25 ± 2 °C and a photoperiod of 16:8 (L:D) h after the initiation of the bioassay. Mortality was evaluated after 24 h. Mortality was assessed by gently touching each individual spider mite with a fine camel hair paint brush under a dissecting stereomicroscope. The individuals with no response were counted as dead. The few moribund individuals that were not able to maintain balance and show uncoordinated twitching were also recorded as dead. The slope, intercept, and LC_50_ (corrected against the untreated control) were evaluated with Abbott’s formula^68^ calculated by log-dose probit analysis (POLO Probit 2014). The statistical analysis of LC_50_ values was based on non-overlapping 95% CI. Resistance ratios (RRs) were calculated through dividing LC_50_ values of field samples by the LC_50_ value of the lab susceptible population.

### Resistance-associated amino acid substitution screening

Genomic DNA was extracted using a DNeasy Blood & Tissue kit (QIAGEN) from 10 adult mites for each population. The DNA was stored at −20 °C till use. The genomic DNA was used as a template for PCR performed in a Peltier-Effect thermal cycler (MJ Research, Inc., Canada). Primers for PCR amplification of regions with resistance-associated point mutations are listed in Table S1. PCR was performed using Phusion High-Fidelity DNA Polymerase (Thermo Scientific, Pittsburgh, PA) under the following cycling parameters: 95 °C for 3 min 50 s, 35 cycles of 94 °C for 35 s, 55 °C for 35 s, and 72 °C for 3 min, with final extension for 10 min at 72 °C. PCR products were purified using DNA Clean & Concentrator (Zymo Research, Irvine, CA) following the manufacturer’s protocol. The purified DNA from each individual was directly sequenced using primers described above (Table S1) for PCR amplification. Each individual PCR product was sequenced using ABI BigDye Terminator Version 3.1 cycle sequencing kit (Applied Biosystems, Foster City, CA) on an ABI 3730 at the Center for Reproductive Biology Molecular Biology and Genomics Core facility at Washington State University. The obtained sequences were analyzed with BioEdit 7.0.1 software (Ibis Biosciences, Carlsbad, CA). The occurrence of mutations was evaluated according to the inspection of sequencing chromatographs, as containing one or both alleles. Each sample was sequenced three times with independently prepared genomic DNAs.

### RNA extraction, cDNA synthesis and qRT-PCR

Total RNA from 100 spider mites per population was extracted using TRIZOL reagent (Invitrogen) following manufacturer’s protocol. The quality of total RNA was checked by gel electrophoresis and spectrometry analyses. The total RNA was treated with DNase I (Ambion Inc., Austin, TX) to remove contaminating DNA. DNase I treated total RNA was used as a template for cDNA synthesizes by M-MLV reverse transcriptase (Promega, Madison, WI). qRT-PCR was performed using a CFX96™ Real-Time PCR Detection System (Bio-Rad Laboratories, Hercules, CA). Each qRT-PCR reaction (10 *μ*l final volume) contained 5 *μ*l iQ™ SYBR Green Supermix (Bio-Rad Laboratories, Hercules, CA), 1.0  *μ*l of cDNA, 3.6 *μ*l ddH_2_O, and 0.4 *μ*l forward and reverse gene specific primers (Table S4, stock 10 *μ*M). An initial incubation of 95 °C for 3  min, followed by 40 cycles of 95 °C for 10 s, 55 °C for 60 s settings were used. The qRT-PCR for each sample was conducted with two technique replicates and three biological replicates. The no-template control and internal controls were included in each plate. *Actin* and *rp49* were used as reference genes for internal controls^49^. Relative expression levels for target genes, in relation to two reference genes, *actin* and *rp49* were calculated by the 2^−∆∆CT^ method^69^. Both the PCR efficiency and R^2^ (correlation coefficient) value were taken into consideration in estimating relative quantities. PCR efficiency between 95% and 105% and R^2^ value >0.99 for each gene were considered as qualified for further analysis.

## Additional Information

**How to cite this article**: Piraneo, T. G. *et al.* Molecular mechanisms of *Tetranychus urticae* chemical adaptation in hop fields. *Sci. Rep.* **5**, 17090; doi: 10.1038/srep17090 (2015).

## Supplementary Material

Supplementary Information

## Figures and Tables

**Table 1:** Baseline toxicity of acaricides in susceptible *T. urticae.*

Acaricide	Field rate (mg a.i./L)	N	LC_50_ (mg a.i./L)	95% CI	Slope ± SEM	Bioassay method	X^2^	df
Abamectin	23	4100	0.228	0.12–0.33	1.87 ± 0.07	Leaf disc	36.02	3
Bifenazate	899	2195	0.820	0.79–0.85	5.69 ± 0.46	Leaf disc	0.12	1
Bifenthrin	120	2300	17.970	8.42–44.60	1.73 ± 0.08	Sticky tape	73.55	3

**Table 2 t2:** Toxicity to abamectin of *T. urticae* populations collected in 2013.

Population	Date	% Mortality^a^	N	LC50 (mg a.i./L)	95% CI	Slope ± SEM	RR	X^2^	df
*Granger1^c^	16 Jul	80.0	180	8.24	6.22–10.73	1.84 ± 0.26	36.14	1.64	2
*Granger 1	20 Aug	92.5	238	7.47	5.77–8.98	3.63 ± 0.60	32.76	3.79	4
*Granger2^d^	16 Jul	100.0	180	2.56	1.82–3.36	2.20 ± 0.29	11.23	1.16	2
*Granger3	25 Jul	74.0	178	9.72	7.48–12.79	2.07 ± 0.33	42.63	1.24	2
*Granger4	25 Jul	92.5	240	1.36	0.15–3.91	0.97 ± 0.13	5.96	11.89	4
*Granger5	25 Jul	95.0	200	8.80	5.59–11.72	4.18 ± 0.66	38.60	3.27	3
*Mabton1^c^	15 Jul	92.0	199	4.24	2.54–6.00	1.69 ± 0.29	18.60	0.65	2
*Moxee1	18 Jul	50.0	160	24.54	11.31–187.00	0.91 ± 0.30	107.63	1.71	2
*Moxee2	29 Aug	77.5	200	13.36	10.95–16.50	3.33 ± 0.63	58.60	0.59	3
**Prosser1**	16 Jun	93.5	276	3.08	0.33–9.52	0.94 ± 0.12	13.51	14.59	4
**Prosser2**	16 Jun	95.0	200	1.94	1.03–2.94	1.36 ± 0.21	8.51	1.94	3
*Prosser2	14 Jul	87.5	200	2.85	1.76–4.04	1.48 ± 0.21	12.50	1.29	3
*Prosser2	28 Jul	89.0	198	7.12	2.12–15.36	2.01 ± 0.25	31.23	9.15	3
**Prosser2**	10 Aug	73.0	198	11.65	6.86–19.41	2.00 ± 0.27	51.10	4.13	3
*Prosser2	19 Aug	75.0	237	–	–	1.69 ± 0.23	–	31.43	4
**Prosser2**	22 Aug	70.0	219	–	–	3.34 ± 0.78	–	9.40	3
*Prosser3	14 Jul	85.0	220	4.97	1.28–9.97	1.68 ± 0.21	21.80	7.47	3
*Prosser3	19 Aug	75.0	220	8.60	5.04–15.69	1.36 ± 0.18	37.72	3.62	4
*Prosser4	17 Jul	62.5	200	11.37	6.01–35.94	1.04 ± 0.26	49.87	1.03	3
*Prosser4	03 Sep	47.5	240	26.05	16.24–62.98	1.28 ± 0.26	114.25	0.33	3
*Prosser 4	08 Sep	54.0	139	–	–	1.54 ± 0.44	–	5.39	2
*Prosser5	24 Jul	92.5	218	8.47	5.98–10.50	2.98 ± 0.69	37.15	1.61	3

^a^% Mortality stands for the % mortality at field rate of abamectin, which is 22.5 mg a.i./L.

^b^RR represents Resistance Ratio = LC_50_ of field population/ LC_50_ of susceptible population.

^c^These populations were reared on lima bean plants for 1 month in the lab prior to bioassay due to the limited spider mite number.

^d^Organic hopyard.

*Molecular data shown in Table 4. -No data available.

**Table 3 t3:** Toxicity to bifenazate of *T. urticae* populations collected in 2013.

Population	Date	N	% Mortality^a^	LC50 (mg a.i./L)	95% CI	Slope ± SEM	RR^b^	X^2^	df
Granger 1	30 Aug	157	82	47.86	11.39 –138.08	1.72 ± 0.22	58.37	3.07	2
*Granger 2^c^	16 Jul	120	100	3.93	0.34–7.11	1.89 ± 0.64	4.79	0.02	1
*Granger 3	20 Aug	197	76	78.97	55.99 –107.50	1.71 ± 0.19	96.30	2.49	3
*Granger 5	25 Jul	160	93	4.88	0.89–10.76	0.99 ± 0.21	5.95	1.87	2
*Mabton 1	27 Jun	60	96	–	–	–	–	–	–
*Mabton 2	27 Jun	60	96	–	–	–	–	–	–
*Moxee 1	18 Jul	160	90	18.88	9.71–30.14	1.38 ± 0.22	23.02	1.95	2
*Prosser 1	14 Jul	60	100	–	–	–	–	–	–
*Prosser 2	28 Jul	160	85	–	–	1.44 ± 0.21	–	3.23	2
Prosser 3	29 Jul	160	88	25.49	3.87–66.98	1.50 ± 0.21	31.09	2.51	2
*Prosser 4	08 Sep	160	90	6.87	1.36–13.66	1.58 ± 0.42	8.38	0.72	2
*Prosser 5	24 Jul	157	95	9.31	3.91–15.69	1.29 ± 0.29	11.35	0.95	2

^a^% Mortality stands for the % mortality at ¼ the field rate of bifenazate, which is 224 mg a.i./L.

^b^RR represents Resistance Ratio = LC_50_ of field population/ LC_50_ of the susceptible population.

^c^Organic hopyard.

*Molecular data shown in Table 4. -No data available.

**Table 4 t4:** Target site mutations in the susceptible and field *Tetranychus urticae* populations for *GluCl1, GluCl3, Cytb*, and *VGSC.*

Population	Date	*GluCl1*(G323D)	*GluCl3*(G326E)	*Cytb*	*VGSC* II	*VGSC* II–III (A1215D)	*VGSC* III (F1538I)
*Susceptible	10 June	G	G	No	No	A	F
Grandview^a^	23 Sep	G	G	–	No	A	I
*Granger 1^a^	16 Jul	G	G	No	No	A	F/I
*Granger 1	20 Aug	G	G	No	No	A	F
*Granger 2^b^	16 Jul	G	G	No	No	A	F
*Granger 3	25 Jul	G	G	No	No	A	F
*Granger 3	20 Aug	G	G	–	No	A	F/I
*Granger 4	25 Jul	G	G	G126G/S	No	A	F/I
*Granger 5	25 Jul	G	G	No	No	A	F/I
Mabton 1	15 Jul	G	G	No	No	A	F/I
*Mabton 1^a^	16 Jul^c^	G	G	No	No	A	F/I
*Mabton 2	15 Jul^c^	G	–	No	No	–	–
Mabton 3	02 Jul	G	G	No	No	A	F/I
*Moxee 1^a^	18 Jul	G	G	G126S	No	A	F
*Moxee 2	29 Aug	G	G	G126S	No	A	F/I
*Prosser 1	14 Jul^c^	G	G	G126G/S	No	A	F/I
*Prosser 2	14 Jul	G	G	No	No	A	F/I
*Prosser 2	28 Jul	G	G	No	No	A	F
*Prosser 2^a^	19 Aug	G	G	No	No	A	F
*Prosser 3	14 Jul	G	G	No	No	A	F
*Prosser 3	19 Aug	G	G	–	No	A	F
*Prosser 4	17 Jul	G	G	–	No	–	I
*Prosser 4	03 Sep	G	G	–	No	A	F/I
*Prosser 4	08 Sep	G	G	G126G/S	No	A	I
*Prosser 5	24 Jul	G	G	G126G/S	No	A	F/I
*Prosser 5	21 Aug	G	G	G126S	No	A	I

^a^Samples were collected for both DNA and RNA extraction.

^b^Organic field.

^c^Spider mite samples were reared on lima bean plants after collection and sampled for DNA extraction one month later.

*Bioassay data shown in Table 1, 2, 3, [Table t2], [Table t2], [Table t2], [Table t3]. No: stands for no mutation identified.-No data available.
