# Supplementary material for: Molecular mechanisms of Tetranychus urticae chemical adaptation in hop fields
Source: Sci Rep. 2015 Dec 1;5:17090. doi: 10.1038/srep17090 (PMC4664861; doi:10.1038/srep17090)
Supplement: Supplementary Information [file srep17090-s1.pdf]

Supplementary material

Title: Molecular mechanisms of *Tetranychus urticae* chemical adaptation in hop fields

Author names: Tara G. Piraneo, Jon Bull, Mariany D. Morales, Laura C. Lavine, Douglas B. Walsh, Fang Zhu*

Supplementary Figure 1. The amino acid substitutions response for abamectin resistance in *T. urticae* and the primer design strategy for TuGlu1 and TuGlu3. TM: transmembran region.

Supplementary Figure 2. The 3D structure for *T. urticae* cytochrome *b* showing amino acid substitutions related to bifenazate resistance. The protein tertiary structure of *T. urticae* cytochrome b was predicted by using the I-TASSER server (http://zhanglab.ccmb.med.umich.edu/I-TASSER/) and then the PDB coordinate file of the highest ranking model was loaded into Chimera (http://plato.cgl.ucsf.edu/chimera/docs/credits.html) for molecular visualization and modification.

Supplementary Figure 3. The structure of sodium channel and tested amino acid substitutions in current study. Mutations labelled with white circles denote that these mutations have been examined in *Xenopus* oocytes and the function in pyrethroid resistance had been confirmed. Mutations labelled with black circles stand for having not been investigated in *Xenopus* oocytes.

Fig. S1

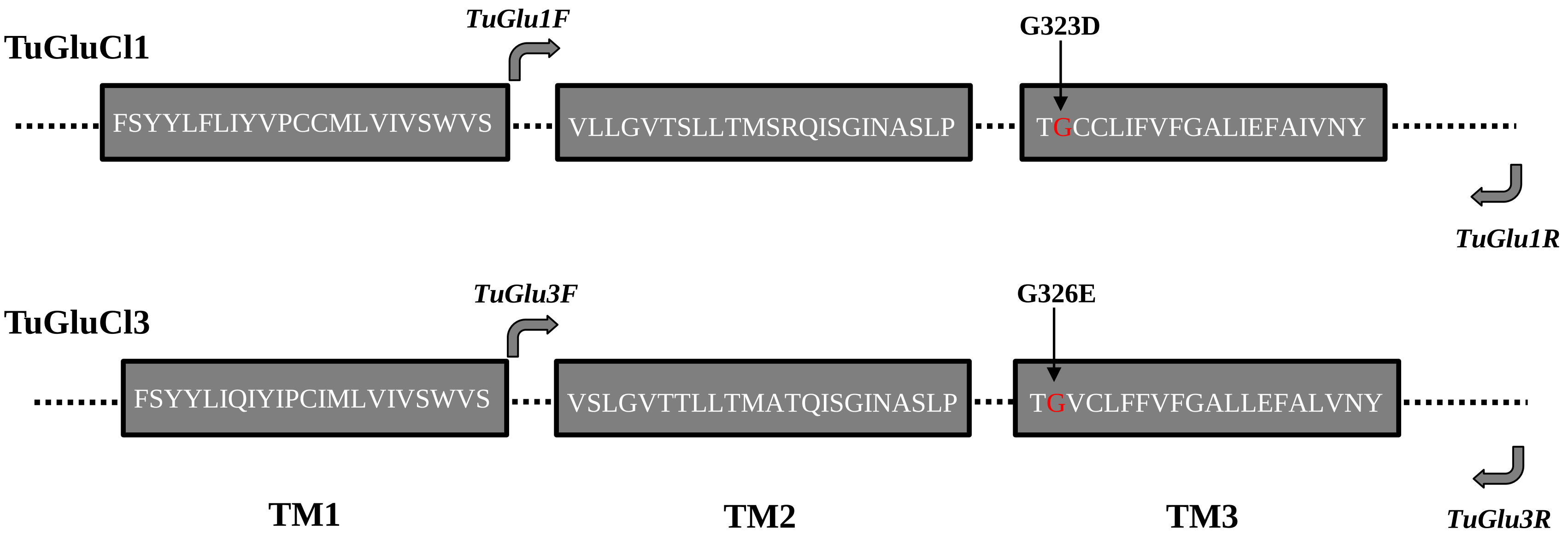
TuGluCl1
TuGlu1F
G323D
FSYYLFLIYVPCCMLVIVSWVS
VLLGVTSLLTMSRQISGINASLP
TGCCLIFVFGALIEFAIVNY
TuGlu1R
TuGluCl3
TuGlu3F
G326E
FSYYLIQIYIPCIMLVIVSWVS
VSLGVTTLLTMATQISGINASLP
TGVCLFFVFGALLEFALVNY
TuGlu3R
TM1
TM2
TM3

Fig. S2

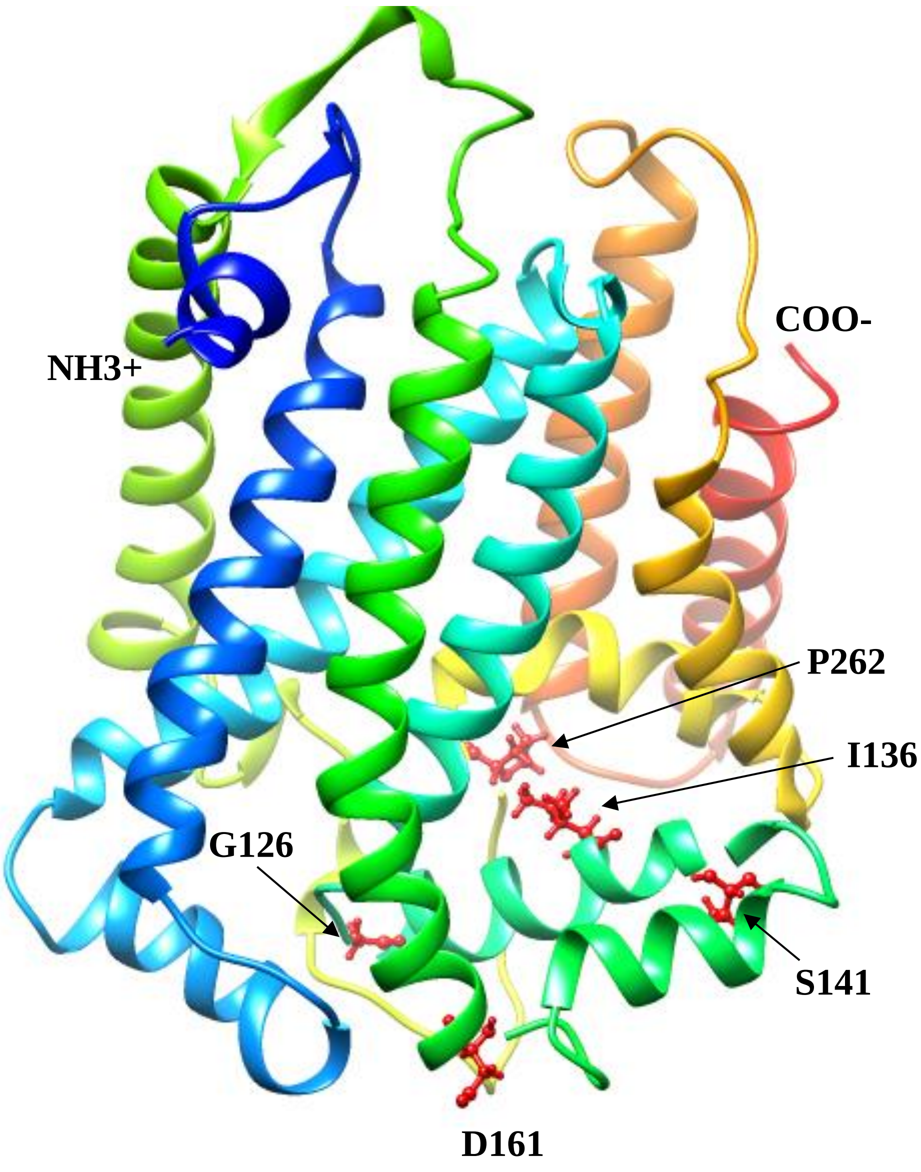

Fig. S3

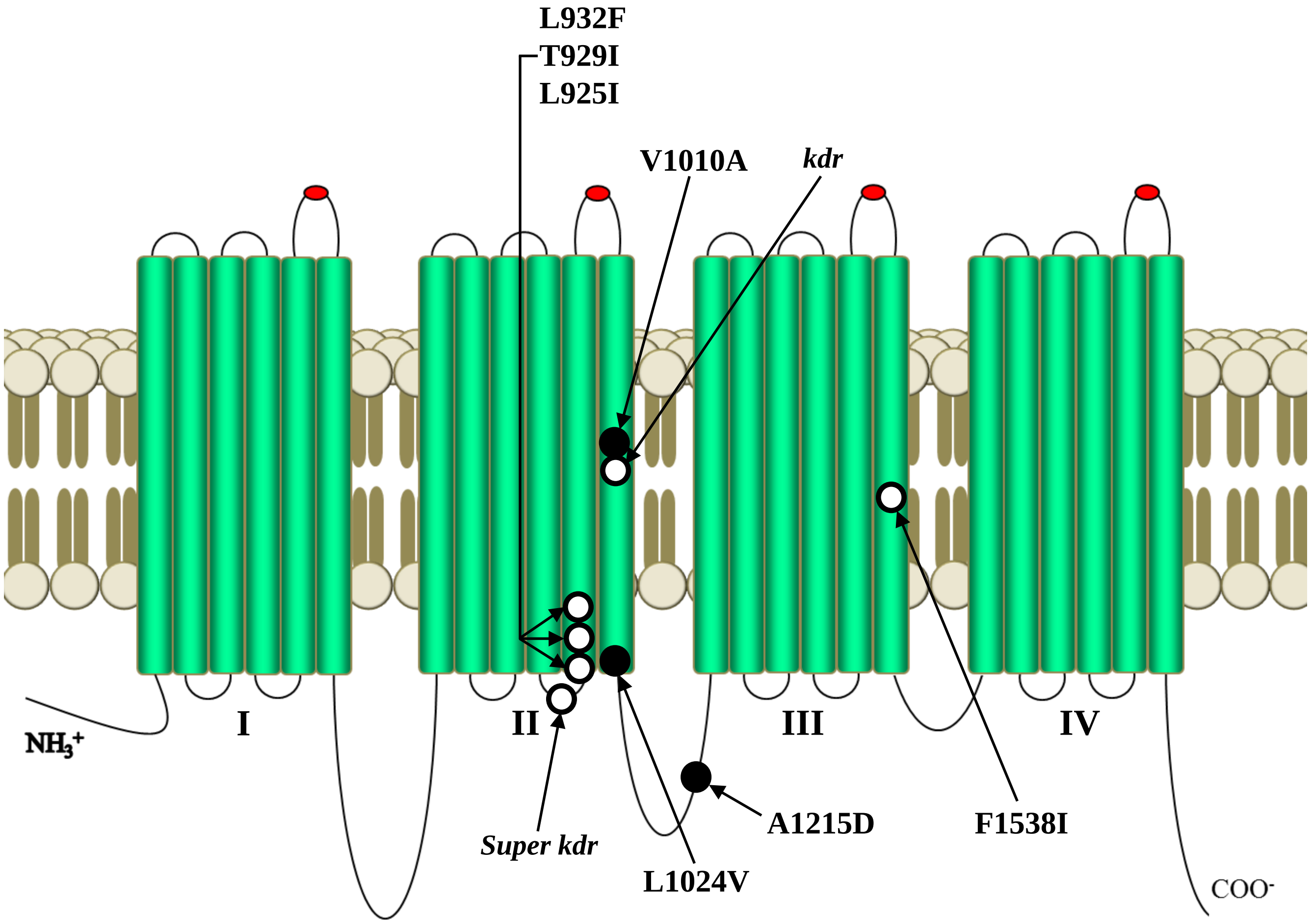

L932F
T929I
L925I
V1010A
kdr
NH3+
I
II
III
IV
Super kdr
L1024V
A1215D
F1538I
COO-

Supplementary Table S1. Primer information for PCR and qRT-PCR analyses

Primer name	Gene name	Gene ID	Primer sequence (5'→ 3')	Amplicon (bp)	Annealing T. (ºC)	Reference
KdrIIF1<br>KdrIIR1	voltage-gated<br>sodium channel	JN881331	F: GAAGGTGTTCGAGGTCTTTCAG<br>R: AAGCGATCAAAGGCTTCTTGAAG	506	55.4<br>55.8	This study
KdrIIF2<br>KdrIIR2	voltage-gated<br>sodium channel	JN881331	F: GTTCGAGGTCTTTCAGTTTTACG<br>R: GGCTTCTTGAAGTTTCTTGGTG	489	53.9<br>54.4	This study
KdrII-IIIF<br>KdrII-IIIR	voltage-gated<br>sodium channel	JN881331	F: TGCATCTCAATTGTCCAAGG<br>R: GTTTCTTCCAGGCAACATGG	255	53.0<br>54.3	*Pest Manag. Sci.*67:1424-1433
KdrIIIF1<br>KdrIIIR1	voltage-gated<br>sodium channel	JN881331	F: TGGACAATTATTATGGACCATGC<br>R: ATGATGGCAGCCAATACACC	1,549	53.2<br>55.5	This study
KdrIIIF1<br>KdrIIIR2	voltage-gated<br>sodium channel	JN881331	F: TGGACAATTATTATGGACCATGC<br>R: GTTTCTTTGATCCCATCTTTTTCAT	259	53.2<br>52.3	This study
TuGlu1F<br>TuGlu1R	glutamate-gated<br>chloride channel 1	AGN53343	F: TTGGATTGACCCTAACTCAGCAG<br>R: TTGCACCAACAATTCCTTGAGG	266	56.8<br>56.1	*Insect Biochem Mol Biol.* 42:455–465
TuGlu3F<br>TuGlu3R	glutamate-gated<br>chloride channel 3	AFG29908	F: CATTTTGGTTGGATCCAAATGCTATTCC<br>R: GTTGAACTTGCTGTTGTTGATTTTCTGCG	267	57.0<br>59.3	This study
CytbF1<br>CytbR1	cytochrome b	EU556749	F: CGGAATAATTTTACAAATAACT<br>R: AATGAAATTTCTGTAAAAGGG	828	51.7<br>50.4	*PNAS* 105 (16): 5980-5985
CytbF1<br>CytbR2	cytochrome b	EU556749	F: CGGAATAATTTTACAAATAACT<br>R: TGGTACAGATCGTAGAATTGC	637	51.7<br>54.6	*PNAS* 105 (16): 5980-5985
TuCYP385C4F<br>TuCYP385C4R	CYP385C4	tetur11g06630	F: CGTAAACGATCGTTCTGGGAAC<br>R: TTTCGGTTTAGGTTTCGGTGAAC	105	55.8<br>55.7	This study
TuCYP389A1F<br>TuCYP389A1R	CYP389A1	tetur25g02050	F: GTTCCTTTCTATTTGGCCATGCG<br>R: TGATCCAGATGGCAAATAAACCCG	135	56.8<br>57.5	This study
TuCYP392D8F<br>TuCYP392D8R	CYP392D8	tetur03g05070	F: CTCGATCATTTCAACGTAAGCCC<br>R: CATGGGCCAGAAGGGAGTTTAC	122	56.0<br>57.8	This study
TuRp49F<br>TuRp49R	ribosomal protein 49	tetur18g03590	F: CTTCAAGCGGCATCAGAGC<br>R: CGCATCTGACCCTTGAACTTC	105	56.6<br>56.0	This study
TuActinF<br>TuActinR	actin	tetur03g09480	F: ATGCCATCCTTCGTTTGGATTTGG<br>R: GGACAATTTCTCGCTCAGCAGTGG	111	58.2<br>58.2	This study
